# Supplementary material for: Drought may exacerbate dryland soil inorganic carbon loss under warming climate conditions
Source: Nat Commun. 2024 Jan 19;15:617. doi: 10.1038/s41467-024-44895-y (PMC10799000; doi:10.1038/s41467-024-44895-y)
Supplement: Supplementary file 1 — Supplementary information [file 41467_2024_44895_MOESM1_ESM.pdf]

Supplementary information for

Drought may exacerbate dryland soil inorganic carbon loss under warming climate conditions

Jinquan Li[1], Junmin Pei[1,2], Changming Fang[1], Bo Li[1,3], Ming Nie[1]

Affiliations:

[1]Ministry of Education Key Laboratory for Biodiversity Science and Ecological Engineering, National Observations and Research Station for Wetland Ecosystems of the Yangtze Estuary, School of Life Sciences, Fudan University, Shanghai, 200438, China.

[2]College of Life Sciences, Shanghai Normal University, Shanghai, 200234, China

[3]Ministry of Education Key Laboratory for Transboundary Ecosecurity of Southwest China, School of Ecology and Environmental Science, Yunnan University, Kunming, 650504, Yunnan, China.

Contents of this file

Supplementary Text

Supplementary Figures 1 to 9

Supplementary Text

Testing the effects of CO_2-free air flushing on Q_{10_SOC} and Q_{10_SIC}

To test the possible effects of CO_2-free air flushing during soil incubation on Q_{10_SOC} and Q_{10_SIC}, we conducted a supplementary experiment. To do this, we examined six representative topsoil (0−10 cm) samples from different aridity areas among the 30 sites; the aridity indices for the six sites where the six representative soils were collected were 0.09, 0.21, 0.32, 0.41, 0.50 and 0.59. Soil samples of 50 g (dry wet) were placed into 250-mL jars and adjusted to 40% water holding capacity. This was conducted for 6 sets for each soil sample (2 flushing treatments (with and without CO_2-free air flushing) × 3 replicates). After a two-week preincubation period at 20°C to minimize disturbances from soil packing and rewatering, the jars with soils were incubated at 5−30°C with a stepwise increase of 5°C to perform the dynamic temperature ramping incubation following the same procedure as described in the Methods in the main text. For the treatment of flushing with CO_2-free air, details of measurements and calculations can be found in the main text. For the treatment of flushing with fresh air, three blank jars (no soil) were also incubated to account for the atmospheric air in the jar, which can dilute the isotopic signature of the emitted $\delta^{13}C_{CO2}$ from the soil. Thus, we conducted a correction using the equations of mass conservation following previous studies[1-3] before the calculations of SOC- and SIC-derived CO_2 emissions and their corresponding ^{13}C isotope values. The results showed that flushing with CO_2-free air enhanced SOC- and SIC-derived CO_2 emissions but had no significant effects on Q_{10_SOC} and Q_{10_SIC} (Supplementary Fig. 8).

Testing the effects of soil sieving on Q_{10_SOC} and Q_{10_SIC}

To test the possible effects of soil sieving on Q_{10_SOC} and Q_{10_SIC}, we conducted a supplementary experiment. To do this, we used six representative topsoil (0−10 cm) samples, which were the same as those used to test the effects of CO_2-free air flushing on Q_{10_SOC} and Q_{10_SIC} (see above). We then

conducted temperature sensitivity assessments for the intact soil (not sieved) and sieved soil (2-mm sieve) from the six representative sites; the temperature sensitivity assessments were conducted following the same procedure as described in the Methods in the main text. The results showed that sieving did not exert significant effects on SOC- and SIC-derived CO_2 emissions and their Q_{10} values (Supplementary Fig. 9).

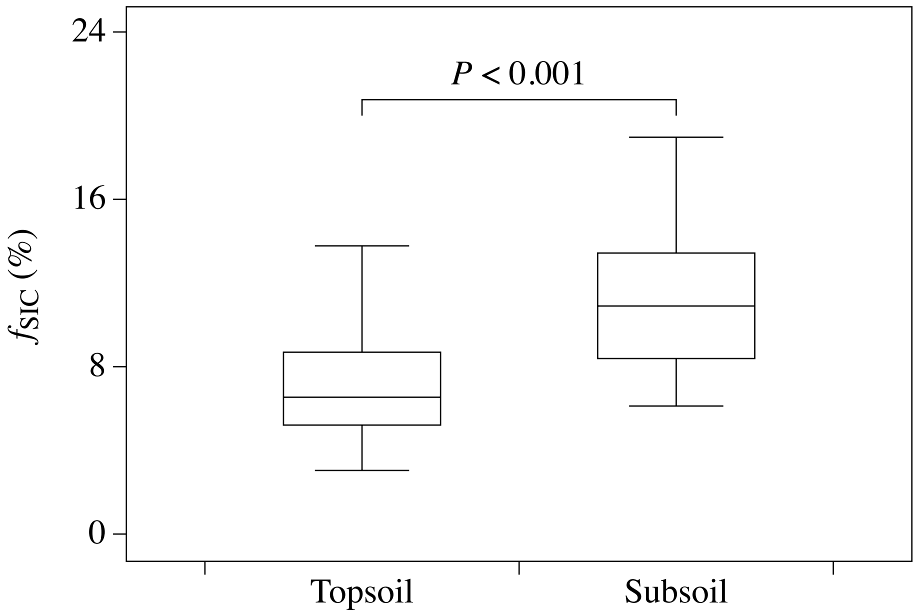

Supplementary Fig. 1 | Differences in the contribution of SIC-derived CO_2 to total CO_2 emissions (f_{SIC}) between the topsoil (0–10 cm) and subsoil (35–50 cm) along an aridity gradient. f_{SIC} was estimated under field moisture conditions at 20°C. The horizontal lines inside the box represent the median, the ends of the boxes represent the first and third quartiles, and the whiskers show the interquartile range from the first and third quartiles. The statistic was derived from $n = 30$ independent samples, and statistical significance was tested using two-sided, paired samples t test.

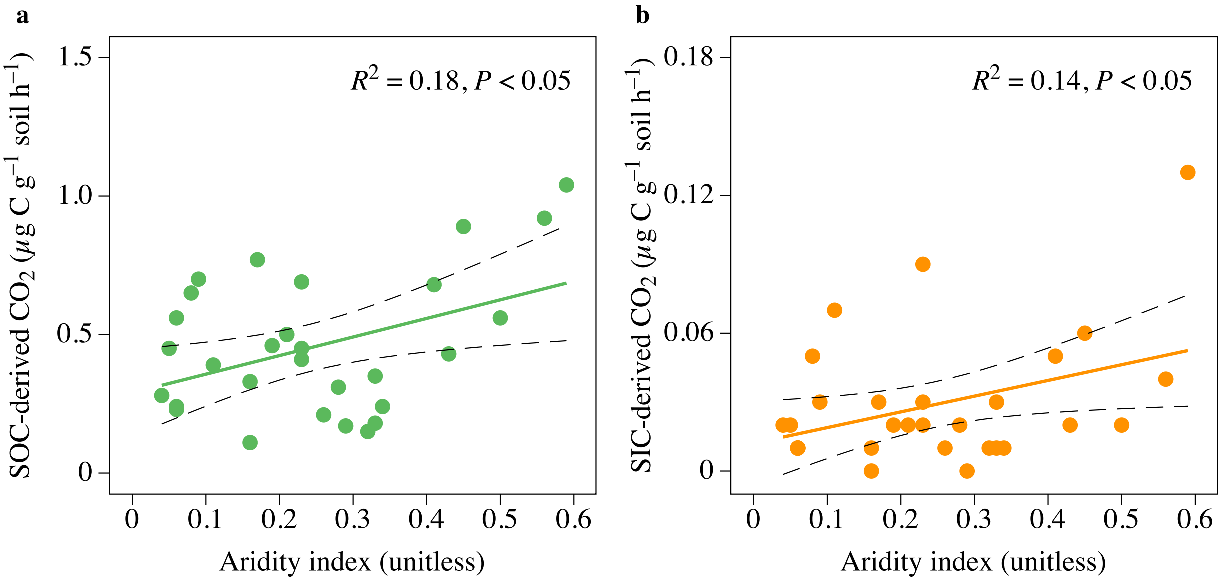

Supplementary Fig. 2 | Changes in SOC-derived and SIC-derived CO_2 emissions in the topsoil along an aridity gradient. a, Linear relationships of SOC-derived CO_2 with the aridity index. **b**, Linear relationships of SIC-derived CO_2 with the aridity index. The dashed lines surrounding the regression lines correspond to the 95% confidence interval of the correlation. SOC-derived and SIC-derived CO_2 emissions were estimated under field moisture conditions at 20°C.

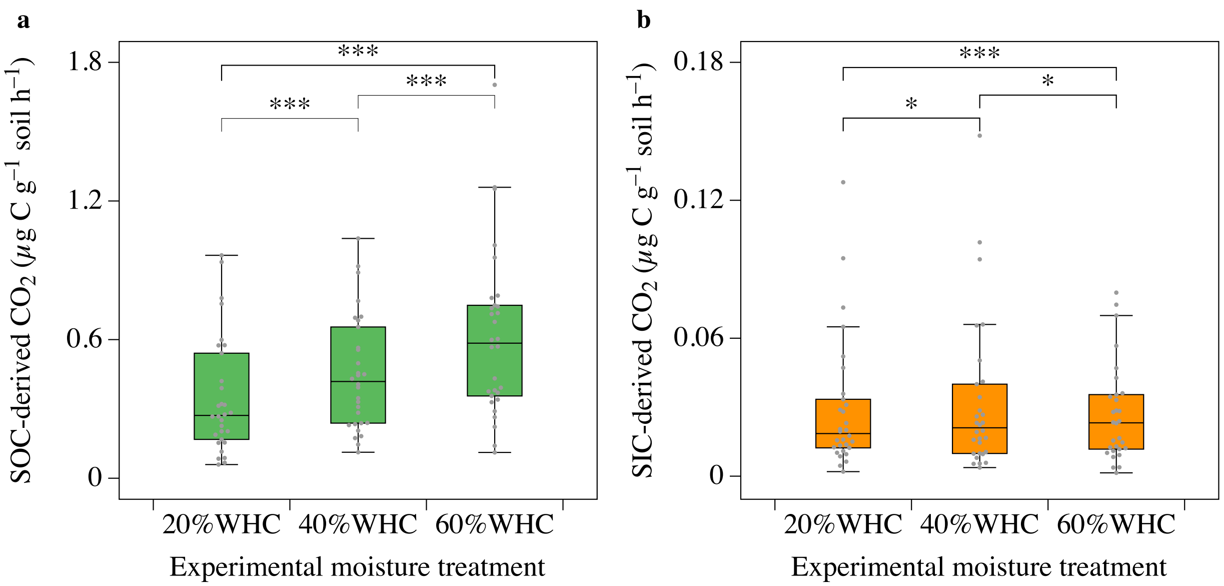

Supplementary Fig. 3 | Differences in SOC- and SIC-derived CO_2 emissions in the topsoil among different experimental moisture treatments. a, Differences in SOC-derived CO_2 among different moisture conditions. **b,** Differences in SIC-derived CO_2 among different moisture conditions. The horizontal lines inside the box represent the median, the ends of the boxes represent the first and third quartiles, and the whiskers show the interquartile range from the first and third quartiles. The gray dots indicate values for each of the 30 sites. All statistics were derived from n = 30 independent samples, and statistical significance was tested using two-sided, paired samples t test. SOC- and SIC-derived CO_2 emissions were estimated at 20°C. *, $P < 0.05$; ***, $P < 0.001$; WHC, water holding capacity.

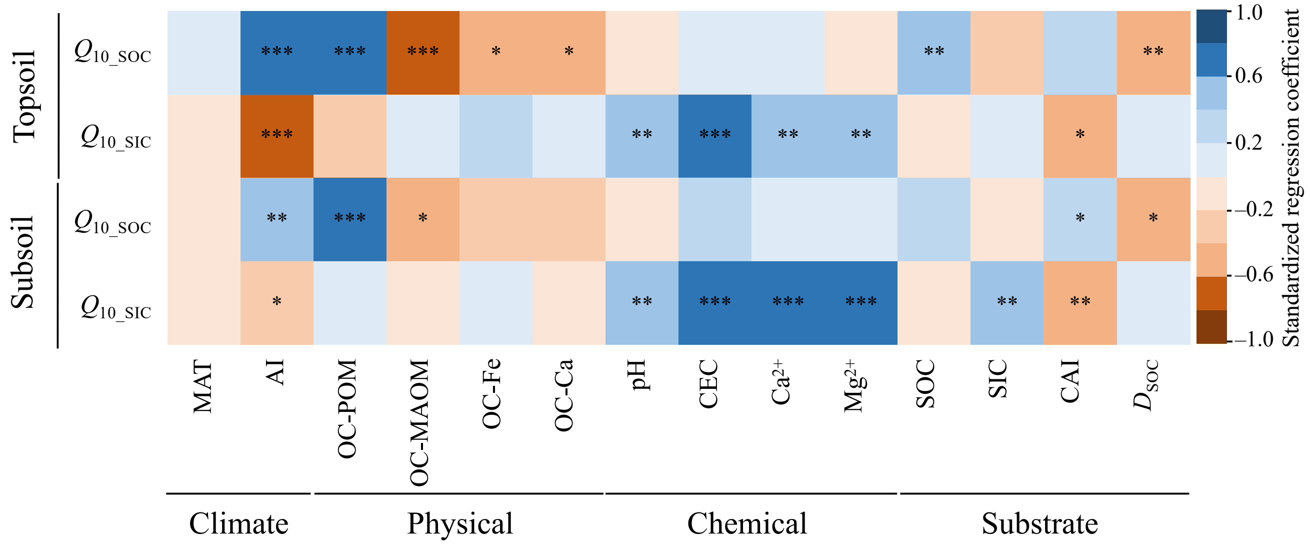

Supplementary Fig. 4 | Pearson correlations (*r*) of the temperature sensitivity (Q_{10}) of SOC- and SIC-derived CO_2 emissions with factors related to climate, physical, chemical and substrate properties. Corrected significance at $P < 0.001$ is represented with ***, $P < 0.01$ is represented with **, and $P < 0.05$ is represented with *. Q_{10} was estimated under a common moisture content of 40% water holding capacity. Q_{10_SOC} and Q_{10_SIC}: Q_{10} of SOC-derived and SIC-derived CO_2 emissions, respectively; MAT: mean annual temperature; AI: aridity index; OC-Ca and OC-Fe, the contents of SOC associated with Ca oxides and Fe bridges, respectively; OC-POM and OC-MAOM, the content of SOC stored in the POM and MAOM fraction, respectively; CEC: cation exchange capacity; CAI, carbon availability index; D_{SOC}, SOC decomposability.

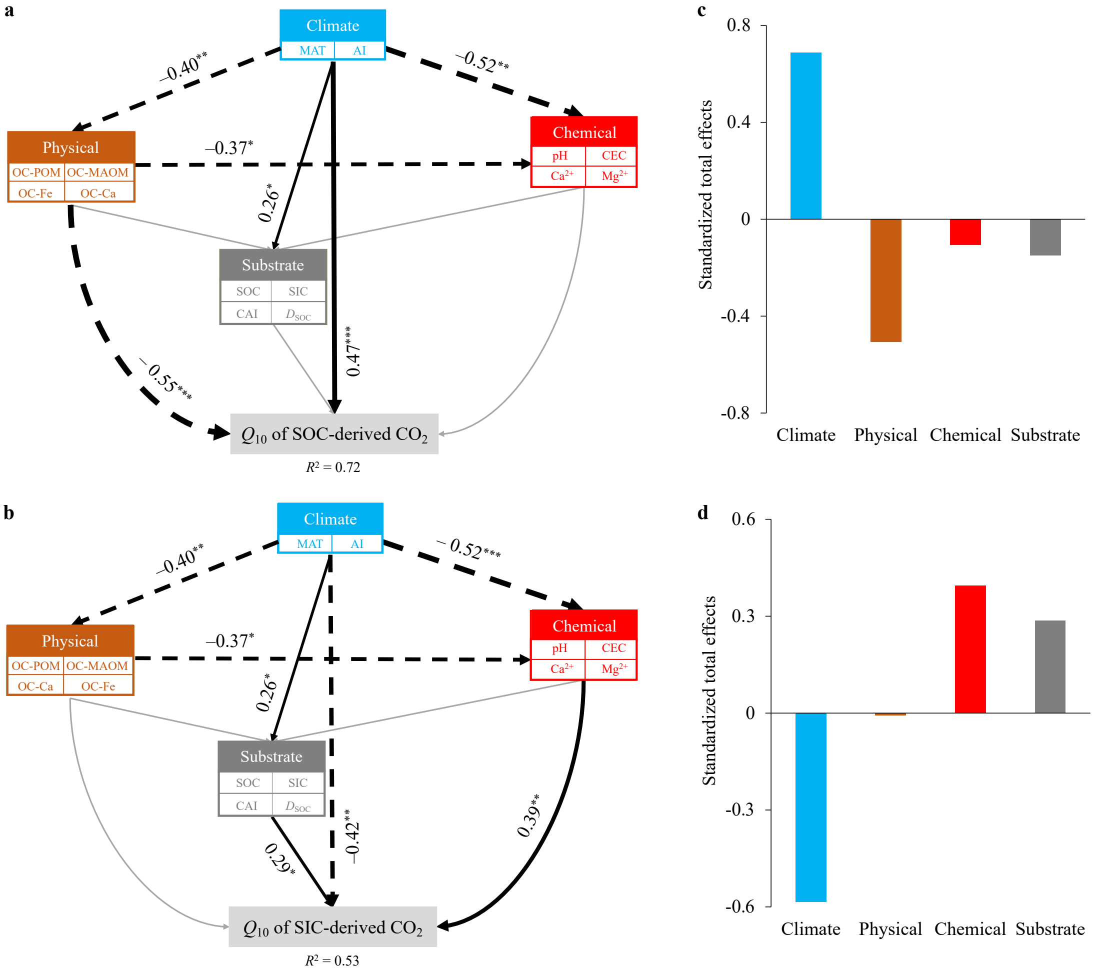

Supplementary Fig. 5 | Direct and indirect effects of climate, physical, chemical and substrate properties on the temperature sensitivity (Q_{10}) of SOC- and SIC-derived CO_2 in the subsoil (35–50 cm). a−b, Structural equation modeling (SEM) was conducted for the Q_{10} of SOC-derived CO_2 (**a**) and SIC-derived CO_2 (**b**). Q_{10} was estimated under field moisture conditions. Black dotted and solid arrows indicate negative and positive relationships, respectively, and gray arrows indicate non-significant relationships; the arrow width represents the strength of the relationship, with the adjacent numbers representing the standardized path coefficients. The multiple-layer rectangles indicate the first component from the principal component analyses conducted for the climate, physical, chemical and substrate properties. **c−d**, The standardized total effects of different factors on Q_{10} of SOC-derived CO_2 (**c**) and SIC-derived CO_2 (**d**) derived from the SEM. MAT: mean annual temperature; AI: aridity index; OC-Ca and OC-Fe, the contents of SOC associated with Ca oxides and Fe bridges, respectively; OC-POM and OC-MAOM, the content of SOC stored in the POM and MAOM fraction, respectively; CEC: cation exchange capacity; CAI, carbon availability index; D_{SOC}, SOC decomposability.

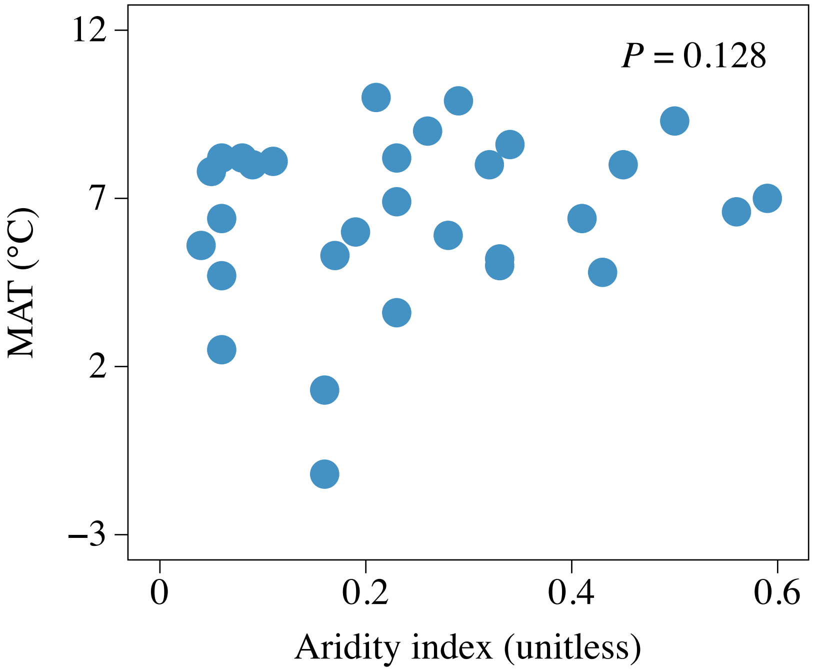

Supplementary Fig. 6 | Relationships of MAT with the aridity index along an aridity gradient in drylands. Linear regression was used. MAT, mean annual temperature.

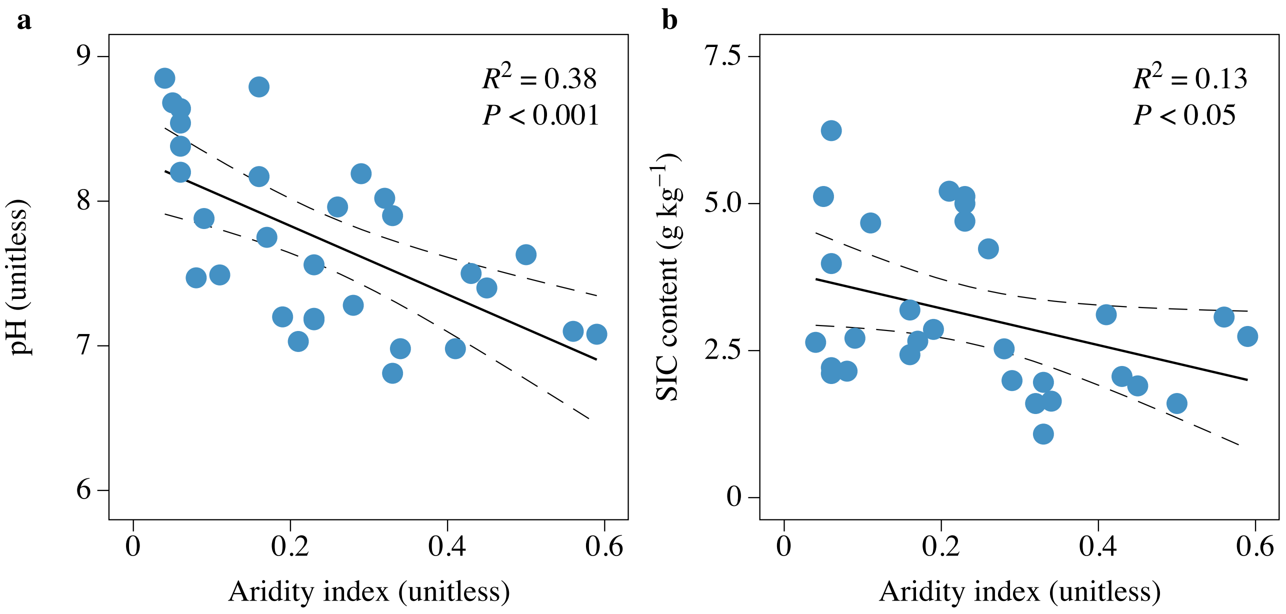

Supplementary Fig. 7 | Changes in soil pH and SIC content along an aridity gradient in drylands. Relationships of soil pH (**a**) and SIC (**b**) with aridity index in the topsoil. Linear regression was used and the dashed lines surrounding the regression lines correspond to the 95% confidence interval of the correlation.

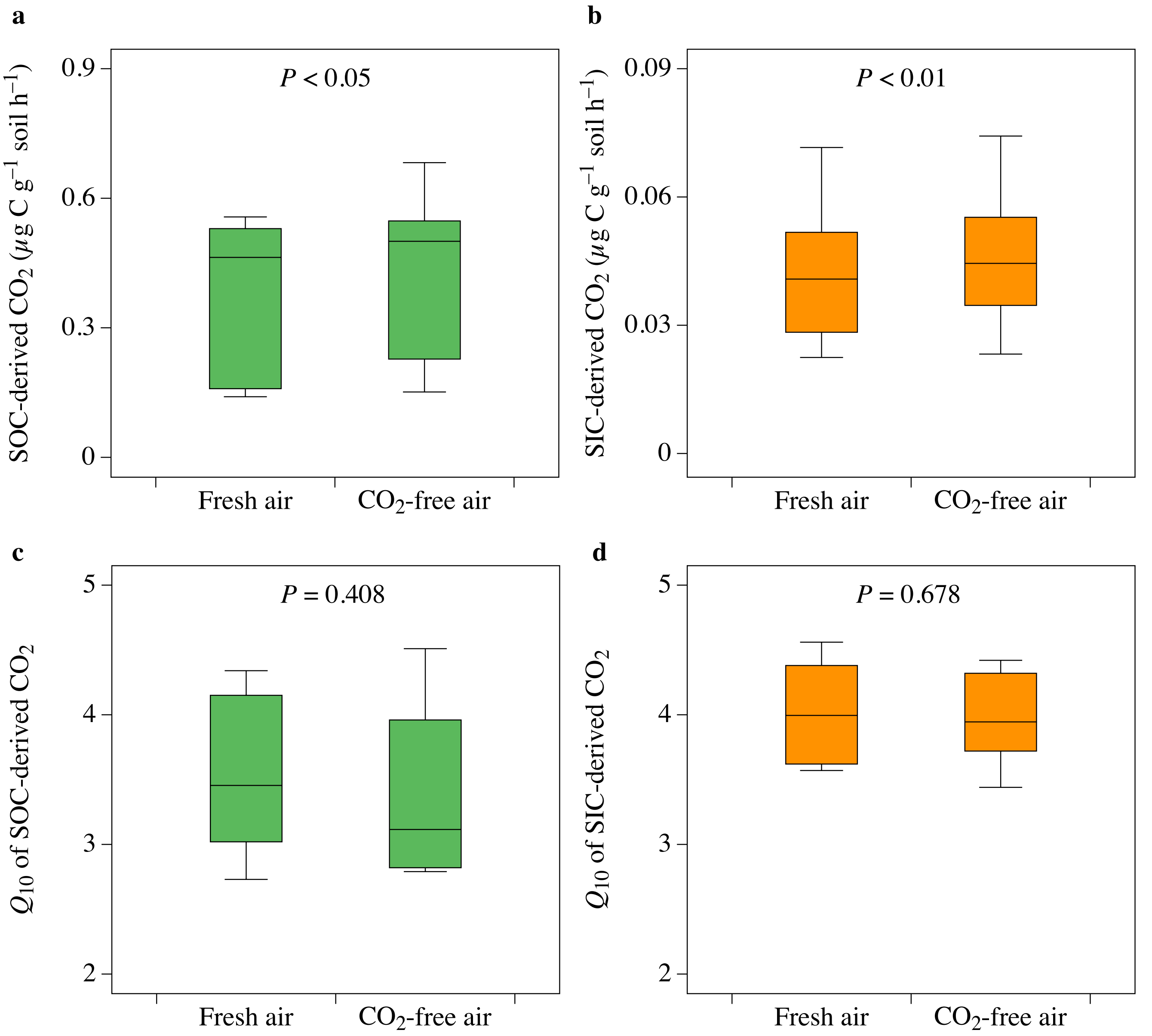

Supplementary Fig. 8 | Differences in SOC- and SIC-derived CO_2 emissions and their temperature sensitivities between the use of fresh air flushing and CO_2-free air flushing. a–b, Differences in SOC-derived (**a**) and SIC-derived (**b**) CO_2 emissions between the use of fresh air flushing and CO_2-free air flushing. **c–d,** Differences in the temperature sensitivity (Q_{10}) of SOC-derived (**c**) and SIC-derived (**d**) CO_2 emissions between the use of fresh air flushing and CO_2-free air flushing. The horizontal lines inside the box represent the median, the ends of the boxes represent the first and third quartiles, and the whiskers show the interquartile range from the first and third quartiles. All statistics were derived from n = 6 independent samples, and statistical significance was tested using two-sided, paired samples t test. SOC- and SIC-derived CO_2 emissions were estimated at 20°C.

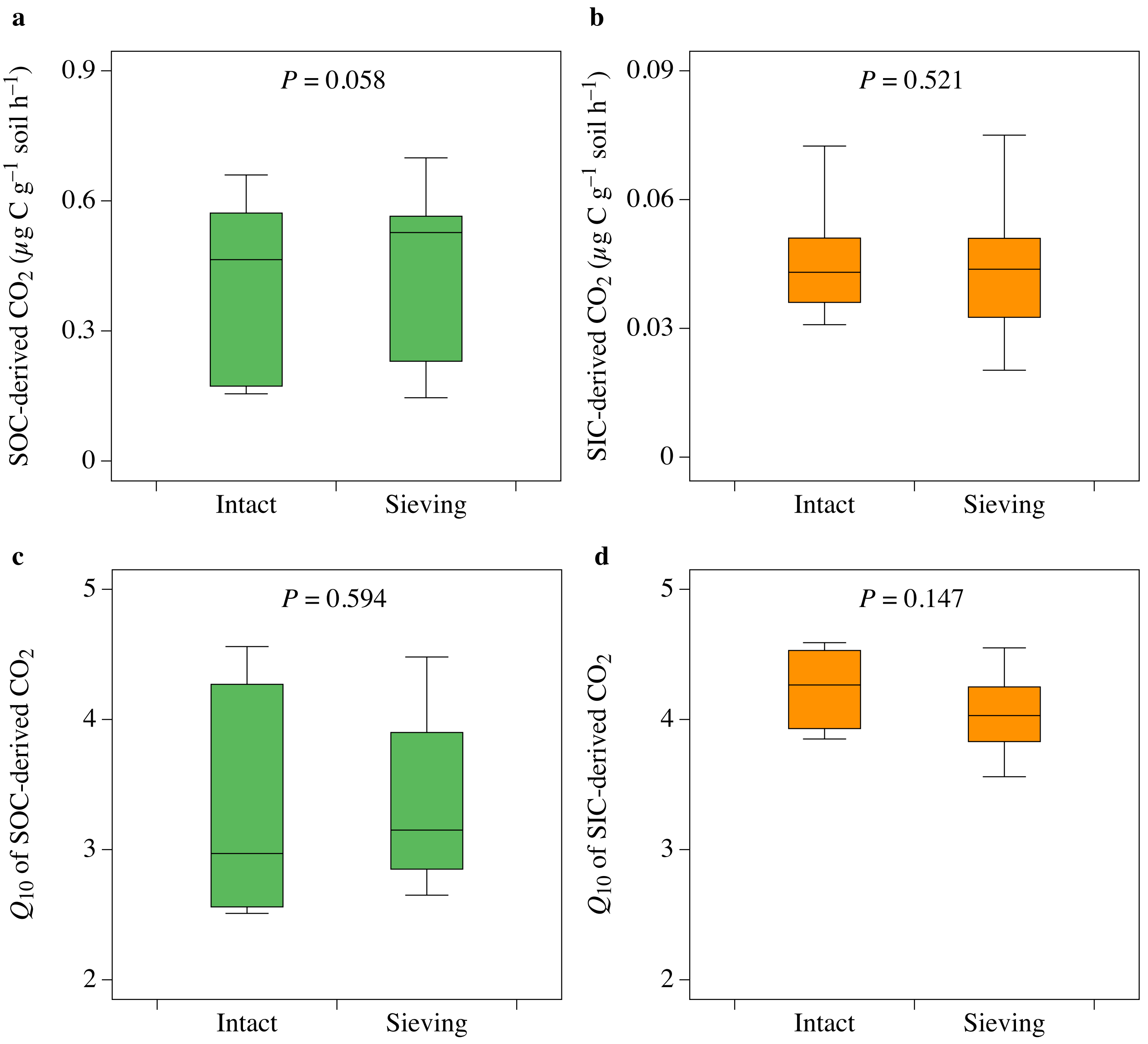

Supplementary Fig. 9 | Differences in SOC- and SIC-derived CO_2 emissions and their temperature sensitivities between intact soil and sieved soil. a–b, Differences in SOC-derived (**a**) and SIC-derived (**b**) CO_2 emissions between intact and sieved soils. **c–d,** Differences in the temperature sensitivity (Q_{10}) of SOC-derived (**c**) and SIC-derived (**d**) CO_2 emissions between intact and sieved soils. The horizontal lines inside the box represent the median, the ends of the boxes represent the first and third quartiles, and the whiskers show the interquartile range from the first and third quartiles. All statistics were derived from n = 6 independent samples, and statistical significance was tested using two-sided, paired samples t test. SOC- and SIC-derived CO_2 emissions were estimated at 20°C.

Supplementary References

1. Tamir, G. et al. Can soil carbonate dissolution lead to overestimation of soil respiration? *Soil Sci. Soc. Am. J.* **75**, 1414−1422 (2011).
2. Yakir, D. & Sternberg, L. d. S. L. The use of stable isotopes to study ecosystem gas exchange. *Oecologia* **123**, 297−311 (2000).
3. Pataki, D. E. et al. The application and interpretation of Keeling plots in terrestrial carbon cycle research. *Glob. Biogeochem. Cycles* **17**, 1022 (2003).
