# Supplementary material for: Drought may exacerbate dryland soil inorganic carbon loss under warming climate conditions
Source: Nat Commun. 2024 Jan 19;15:617. doi: 10.1038/s41467-024-44895-y (PMC10799000; doi:10.1038/s41467-024-44895-y)
Supplement: Supplementary file 3 — Reporting Summary [file 41467_2024_44895_MOESM3_ESM.pdf]

nature portfolio

Corresponding author(s): Ming Nie

Last updated by author(s): Dec 27, 2023

Reporting Summary

Nature Portfolio wishes to improve the reproducibility of the work that we publish. This form provides structure for consistency and transparency in reporting. For further information on Nature Portfolio policies, see our Editorial Policies and the Editorial Policy Checklist.

Statistics

For all statistical analyses, confirm that the following items are present in the figure legend, table legend, main text, or Methods section.

n/a	Confirmed	
☐	☒	The exact sample size (*n*) for each experimental group/condition, given as a discrete number and unit of measurement
☐	☒	A statement on whether measurements were taken from distinct samples or whether the same sample was measured repeatedly
☐	☒	The statistical test(s) used AND whether they are one- or two-sided<br>*Only common tests should be described solely by name; describe more complex techniques in the Methods section.*
☒	☐	A description of all covariates tested
☐	☒	A description of any assumptions or corrections, such as tests of normality and adjustment for multiple comparisons
☐	☒	A full description of the statistical parameters including central tendency (e.g. means) or other basic estimates (e.g. regression coefficient) AND variation (e.g. standard deviation) or associated estimates of uncertainty (e.g. confidence intervals)
☐	☒	For null hypothesis testing, the test statistic (e.g. *F*, *t*, *r*) with confidence intervals, effect sizes, degrees of freedom and *P* value noted<br>*Give P values as exact values whenever suitable.*
☒	☐	For Bayesian analysis, information on the choice of priors and Markov chain Monte Carlo settings
☒	☐	For hierarchical and complex designs, identification of the appropriate level for tests and full reporting of outcomes
☐	☒	Estimates of effect sizes (e.g. Cohen's *d*, Pearson's *r*), indicating how they were calculated

Our web collection on statistics for biologists contains articles on many of the points above.

Software and code

Policy information about availability of computer code

Data collection	No software was used to collect data in this study.
Data analysis	Statistical analyses were performed using the SPSS version 23 (IBM SPSS Statistics for Windows, Version 23.0. IBM Corp., Armonk, NY, USA). The structural equation model was conducted using AMOS 21.0 software (Amos Development Corporation, Chicago, IL).

For manuscripts utilizing custom algorithms or software that are central to the research but not yet described in published literature, software must be made available to editors and reviewers. We strongly encourage code deposition in a community repository (e.g. GitHub). See the Nature Portfolio guidelines for submitting code & software for further information.

Data

Policy information about availability of data

All manuscripts must include a data availability statement. This statement should provide the following information, where applicable:

- Accession codes, unique identifiers, or web links for publicly available datasets
- A description of any restrictions on data availability
- For clinical datasets or third party data, please ensure that the statement adheres to our policy

MAT and MAP data were obtained from the WorldClim (https://www.worldclim.org/). Aridity indices were obtained from the Global Aridity Index and Potential Evapotranspiration Climate database (https://cgiarcsi.community/). The Q10 value and soil properties data are available at https://doi.org/10.5281/zenodo.10370941

Research involving human participants, their data, or biological material

Policy information about studies with human participants or human data. See also policy information about sex, gender (identity/presentation), and sexual orientation and race, ethnicity and racism.

Reporting on sex and gender	This study is not related to human research.
Reporting on race, ethnicity, or other socially relevant groupings	This study is not related to human research.
Population characteristics	This study is not related to human research.
Recruitment	This study is not related to human research.
Ethics oversight	This study is not related to human research.

Note that full information on the approval of the study protocol must also be provided in the manuscript.

Field-specific reporting

Please select the one below that is the best fit for your research. If you are not sure, read the appropriate sections before making your selection.

☐ Life sciences ☐ Behavioural & social sciences ☒ Ecological, evolutionary & environmental sciences

For a reference copy of the document with all sections, see nature.com/documents/nr-reporting-summary-flat.pdf

Ecological, evolutionary & environmental sciences study design

All studies must disclose on these points even when the disclosure is negative.

Study description	To reveal the general effects and mechanisms of moisture on Q10_SOC and Q10_SIC , we conducted two experiments (Fig. 1): a natural aridity gradient and a moisture control treatment. In the first experiment, soil moisture regime differences were evaluated by sampling soils from 30 sites across a wide aridity index (ranging from 0.04 to 0.59) along an approximately 4,500 km east–west transect in the drylands of northern China; in this experiment, Q10_SOC and Q10_SIC were determined with field moisture conditions. In the second experiment, to directly test the effects of only moisture changes on Q10_SOC and Q10_SIC, we conducted a moisture control experiment by incubating soils under different moisture conditions at 20%, 40% and 60% water holding capacity (WHC). To determine the main drivers and their differences associated with variations in Q10_SOC and Q10_SIC that were determined under field moisture conditions along the aridity gradient, we analyzed various potential factors related to climate (MAT and aridity index), physical (OC-POM and OC-MAOM fractions, and OC-Ca and OC-Fe), chemical (pH, CEC, Ca2+ and Mg2+) and substrate (quantity, quality and availability) properties.
Research sample	For the first experiment, soils (topsoil of 0–10 cm and subsoil of 35–50 cm) were collected from 30 sites along an approximately 4,500 km east–west transect with a longitudinal gradient of 81.02–123.53°E in northern China (Fig. 1), and soils with field moisture conditions were further used. For the second experiment, soils from the 30 sites were adjusted to different moisture conditions of 20%, 40% and 60% WHC, and soils with different manipulated moisture conditions were further used.
Sampling strategy	Three random locations (greater than 20 m apart from each other) were chosen at each site. At each site, soils from different depths of the topsoil (0–10 cm) and subsoil (35–50 cm) were collected. As our aim was to reveal the general effects of moisture on Q10 of SOC and SIC, not to assess its variability within each sites, soils from the three random locations were gently mixed to produce a homogeneous composite sample for each depth at each site.
Data collection	The original data of this study was collected mainly by Jinquan Li and Junmin Pei, with the help from graduate student of Xinxin Xu. The CO2 concentration and δ13C value of gas samples were analyzed using a gas isotope analyzer (G2201-20i, Picarro, USA). For soil physical properties, a fractionation technique was adopted to estimate the SOC stored in the POM and MAOM fractions. For soil chemical properties, Ca2+ and Mg2+ contents were measured by using inductively coupled plasma–optical emission spectrometry, and CEC was determined by using a microplate reader (Synergy 2, BioTek, USA). For substrate properties, SOC content was analyzed using an elemental analyzer (Multi EA 4000, Analytik Jena, Germany), and SIC was determined by a pressure calcimeter method.
Timing and spatial scale	The aim of this study was to reveal the general effects of moisture on Q10 of SOC and SIC. To do so, soils were collected from 30 sites along an approximately 4,500 km east–west transect in northern China. Incubation experiments for the first experiment (the aridity gradient) were conducted from May to September 2020; incubation experiments for the second experiment (a soil moisture control experiment) were conducted from July to October 2021. Soil physical, chemical and substrate properties were determined from May to December 2020 and from July to December 2021.
Data exclusions	No data were excluded from this study.
Reproducibility	In the whole experiment, all the findings can be replicated as all the incubation and measurement techniques are widely used and can be reproduced. Four technical replicates were conducted for soil incubations, and three technical replicates were conducted for

soil physical, chemical and substrate property measurements.

Randomization	For soil sampling, three random locations (more than 20 m apart from each other) were chosen at each site. The soil samples were passed through a 2-mm sieve and soils from the three random locations were gently mixed to produce a homogeneous composite sample for each depth at each site. For soil incubation for both experiments, 50 g of dry-weight fresh soil, with four experimental replicates, were incubated in 250 ml jars. The order of measurements for the four experimental replicates was fully randomized.
Blinding	Our graduate research assistants had no idea what the bottle labels meant.

Did the study involve field work? ☒ Yes ☐ No

Field work, collection and transport

Field conditions	Soils were collected from 30 sites along an approximately 4,500 km east–west transect in northern China. The MAT and MAP ranged from −1.2 to 10.0°C and from 46 to 486 mm, respectively.
Location	The longitudes of the 30 sites ranged from 81.02 to 123.53°E in northern China.
Access & import/export	No permit was required. Soil samples were sealed in sterile polypropylene bags and transported to laboratory using iceboxes.
Disturbance	To minimize the effects of our sampling, we backfill the soil profiles.

Reporting for specific materials, systems and methods

We require information from authors about some types of materials, experimental systems and methods used in many studies. Here, indicate whether each material, system or method listed is relevant to your study. If you are not sure if a list item applies to your research, read the appropriate section before selecting a response.

Materials & experimental systems

n/a	Involved in the study	
☒	☐	Antibodies
☒	☐	Eukaryotic cell lines
☒	☐	Palaeontology and archaeology
☒	☐	Animals and other organisms
☒	☐	Clinical data
☒	☐	Dual use research of concern
☒	☐	Plants

Methods

n/a	Involved in the study	
☒	☐	ChIP-seq
☒	☐	Flow cytometry
☒	☐	MRI-based neuroimaging
